# Breakthrough Infections with Multiple Lineages of SARS-CoV-2 Variants Reveals Continued Risk of Severe Disease in Immunosuppressed Patients

**DOI:** 10.3390/v13091743

**Published:** 2021-09-01

**Authors:** Xufang Deng, Monika Evdokimova, Amornrat O’Brien, Cynthia L. Rowe, Nina M. Clark, Amanda Harrington, Gail E. Reid, Susan L. Uprichard, Susan C. Baker

**Affiliations:** 1 Department of Microbiology and Immunology, Stritch School of Medicine, Loyola University Chicago, Chicago, IL 60153, USA; xufang.deng@okstate.edu (X.D.); mevdokimova@luc.edu (M.E.); aobrien6@luc.edu (A.O.); cynthiarowe@comcast.net (C.L.R.); suprichard@luc.edu (S.L.U.); 2 Department of Medicine, Stritch School of Medicine, Loyola University Chicago, Chicago, IL 60153, USA; NMCLARK@lumc.edu (N.M.C.); greid@lumc.edu (G.E.R.); 3 Infectious Disease and Immunology Research Institute, Stritch School of Medicine, Loyola University Chicago, Chicago, IL 60153, USA; Amanda.Harrington@lumc.edu; 4 Department of Pathology and Laboratory Medicine, Stritch School of Medicine, Loyola University Chicago, Chicago, IL 60153, USA

**Keywords:** SARS-CoV-2, variants of concern, breakthrough infections, post-vaccination infections, COVID-19, immunosuppression

## Abstract

The pandemic of COVID-19 caused by SARS-CoV-2 infection continues to spread around the world. Vaccines that elicit protective immunity have reduced infection and mortality, however new viral variants are arising that may evade vaccine-induced immunity or cause disease in individuals who are unable to develop robust vaccine-induced responses. Investigating the role of viral variants in causing severe disease, evading vaccine-elicited immunity, and infecting vulnerable individuals is important for developing strategies to control the pandemic. Here, we report fourteen breakthrough infections of SARS-CoV-2 in vaccinated individuals with symptoms ranging from asymptomatic/mild (6/14) to severe disease (8/14). High viral loads with a median C*t* value of 19.6 were detected in the nasopharyngeal specimens from subjects regardless of disease severity. Sequence analysis revealed four distinct virus lineages, including *alpha* and *gamma* variants of concern. Immunosuppressed individuals were more likely to be hospitalized after infection (*p* = 0.047), however no specific variant was associated with severe disease. Our results highlight the high viral load that can occur in asymptomatic breakthrough infections and the vulnerability of immunosuppressed individuals to post-vaccination infections by diverse variants of SARS-CoV-2.

## 1. Introduction

COVID-19 vaccine deployment has dramatically reduced the number of infections, hospitalizations, and deaths caused by SARS-CoV-2 [1,2]. However, there are reports of SARS-CoV-2 infections in fully vaccinated individuals [3,4,5,6,7,8,9,10,11,12,13,14,15,16]. In the majority of individuals, these infections are presumed to be breakthrough infections associated with SARS-CoV-2 variants that escape vaccine-induced immunity. Indeed, studies have experimentally shown that serum from vaccinated individuals is less efficient in neutralizing viral variants [17,18]. On the other hand, post-vaccination infection may occur if vaccine-induced immunity is absent or ineffective, such as in solid organ transplant (SOT) recipients who are receiving immunosuppressive therapy [19,20,21,22,23,24]. Here we describe the characteristics of fourteen SARS-CoV-2 infections in fully vaccinated individuals at our medical center between 29 March 2021 and 29 April 2021 that represent both of these scenarios.

## 2. Materials and Methods

### 2.1. Specimen Collection and Processing

Between 29 March 2021 and 29 April 2021 we collected nasopharyngeal swab samples from all vaccinated individuals who tested SARS-CoV-2 positive. The protocol for the collection and banking of de-identified samples and associated clinical data was reviewed by the Loyola University Health Sciences Campus Institutional Review Board (IRB) and deemed exempt in accordance with the Department of Health and Human Services regulations at 45 CFR 46.104(d)(4)(ii-iii)(IRB 214365). The de-identified samples and data were then distributed for research under IRB 214451. The demographic and clinical data from the 14 subjects who tested SARS-CoV-2 positive in this one-month period are shown in Table 1. A five hundred microliter sample from the nasopharyngeal swab specimen was processed for RNA extraction.

### 2.2. Detection of Viral RNA and Cycle Threshold (Ct)

Nasopharyngeal swab specimens were extracted for RNA using MagMAX™ Viral RNA Isolation Kit (ThermoFisher Scientific, Carlsbad, CA, USA) following the manufacturer’s instruction. The isolated RNA was eluted in 50 μL nuclease-free water and 5 μL was used for TaqPath 1-Step RT-PCR (ThermoFisher Scientific, Carlsbad, CA, USA) to measure the level of viral nucleocapsid gene with primers (N1 Forward: 5′-GACCCCAAAATCAGCGAAAT-3′; N1 Reverse: 5′-TCTGGTTACTGCCAGTTGAATCTG-3′) and probe (FAM-ACCCCGCAT /ZEN/ TACGTTTGGTGGACC-3IABkFQ). 

### 2.3. Sequencing of SARS-CoV-2 Complete Genome

Complete genome amplification using the ARTIC network V3 multiplex primers was performed followed by next-generation sequencing [25]. Briefly, reverse transcription of RNA samples was completed with LunaScript™ RT SuperMix Kit (NEB) according to the manufacturer’s instructions. Multiplexing PCR amplification of cDNA was performed using Q5 Hot Start High-Fidelity Master mix (NEB) with the ARTIC network V3 multiplex primers. Equal molar weight of DNA products of two multiplexing PCR were pooled and subjected to a high-throughput amplicon sequencing (Genewiz, NJ, USA). Sequencing reads were quality controlled by removing low quality reads (Q score < 20) and trimming primer sequences. Trimmed reads were aligned to the SARS-CoV-2 reference genome using a bioinformatic tool, iVar [26]. Consensus sequence was obtained when minimal coverage reached 50 (*p* < 0.05). Complete genomic sequences were analyzed with the NextClade online software (https://clades.nextstrain.org, accessed on 21 July 2021) to assign the clade and call amino acid mutations. 

### 2.4. Evaluating Antibody Response to SARS-CoV-2 Spike Protein

Enzyme-linked immunosorbent assay (ELISA) was performed using the spike antigen (hexapro) [27], according to the method described by Stadlbauer et al. [28]. Serial dilutions of patient serum were evaluated to identify the point where absorbance was at least 3-fold over the OD of the control serum, which was designated as the end-point titer.

### 2.5. Statistical Analysis

Binomial logistic regression analyses were performed using the R program to examine the association between explanatory variables and a response variable (hospitalization). 

## 3. Results

We describe SARS-CoV-2 infections over a 4-week period from 29 March 2021 to 29 April 2021 in fourteen fully vaccinated individuals at Loyola University Medical Center (LUMC) in suburban Chicago, Illinois (Table 1). These breakthrough infections represent 2% of the 692 positive tests, from a total of 9752 samples evaluated during this time period. The subjects ranged in age from 37 to 81 years old (mean = 60.3) with eight females and six males, 3/14 were healthcare workers and 6/14 were solid organ transplant (SOT) recipients. All subjects had documented records of completing their vaccine regimens at least 14 days prior to testing positive for COVID-19 (range: 14 to 109 days). Three subjects were identified by surveillance or post-exposure testing (S1, S5, and S6) and reported either no symptoms or only rhinorrhea. Serum obtained from Subject 1 at day 2 after testing positive for SARS-CoV-2 revealed a high titer anti-spike antibody response (1:40,000 endpoint titer), which increased by day 24 after infection (1:80,000 endpoint titer), consistent with a boosted anti-spike response after breakthrough infection. Three subjects (S2, S3, and S4) reported symptomatic COVID-19 but did not require hospitalization. Eight subjects (S7–S14) were hospitalized with COVID-19, with four requiring admission to the intensive care unit (ICU). Thirteen subjects recovered from COVID-19; one succumbed to infection. Statistical analysis indicates that being elderly (age > 60) (*p* = 0.0202) and ongoing treatment with immunosuppressive medication (*p* = 0.047) was significantly associated with severe disease requiring hospitalization after infection. Six of the hospitalized COVID-19 patients were solid organ transplant (SOT) recipients who had completed their vaccine regimens (S8–S13).

To evaluate the viral load in these subjects, RNA was isolated from the nasopharyngeal swabs of all 14 subjects and evaluated for the presence of SARS-CoV-2 RNA using TaqPath 1-step RT-PCR. The cycle threshold (Ct) values ranged from 15.7 to 34.2 (median = 19.6). Only one patient, a healthcare worker without other apparent risks for COVID-19, had a C*t* > 30. There was no association found between disease severity and viral load (*p* = 0.45), with the average C*t* of the non-hospitalized versus hospitalized group being 19.2 versus 19.6, respectively. These results document the high viral loads present in both symptomatic and asymptomatic patients after breakthrough infections.

**Figure 1:**
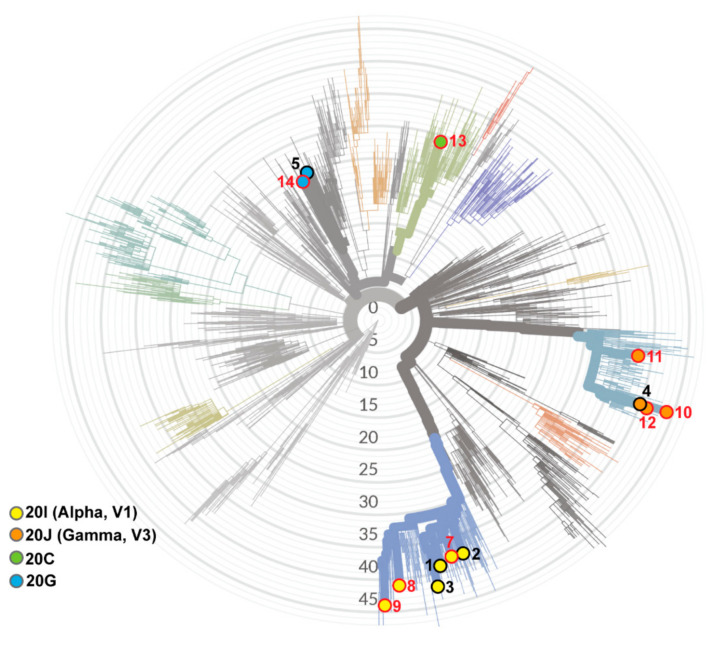
Sequence analyses reveal multiple lineages of SARS-CoV-2 variants associated with breakthrough infections in this study. Phylogenetic tree depicting the lineages of the thirteen SARS-CoV-2 genomic sequences. The complete genome nucleotide sequence obtained from each subject was analyzed with NextClade software (https://clades.nextstrain.org/tree, accessed on 21 July 2021) for clade assignment. WHO designation of variants of concern (VOC) are given in parenthesis. Hospitalized subjects are indicated with a red circle and number; subjects with mild/asymptomatic disease are indicated with a black circle and number.

**Figure 2:**
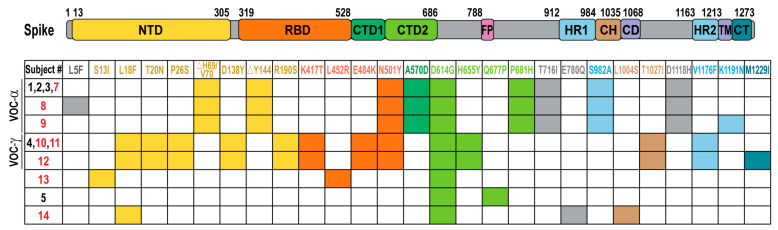
Multiple mutations in the SARS-CoV-2 spike gene were identified in subjects with breakthrough infections. Schematic diagram of the SARS-CoV-2 spike with functional domains highlighted in color. NTD: N-terminal domain; RBD: receptor-binding domain; CTD1 and CTD2: C-terminal domain 1 and 2; FP: fusion peptide; HR1: heptad repeat 1; CH: central helix; CD: connector domain; HR2: heptad repeat 2; TM: transmembrane domain; CT: cytoplasmic tail. The amino acid substitutions deduced from the nucleotide sequence detected in each subject are shown in the boxes and colored according to their location in the spike sequence. Subject numbers in red are the hospitalized cases.

To identify the lineage of the SARS-CoV-2 in these patients, we performed the ARTIC multiplexing PCR and high-throughput amplicon sequencing of each sample. Thirteen complete genomes were obtained from the samples with C*t* < 30, with an average coverage above 2000 (2009–3381). Phylogenetic analysis of the complete genomic sequences using the NextClade online software revealed four distinct lineages of SARS-CoV-2 (Figure 1). The majority of the subjects were infected with variants belonging to the *Alpha* lineage (also known as B.1.1.7) (6/13) or the *Gamma* lineage (also known as P.1 variant) (4/13), which were the most common variants of concern circulating in the USA in April of 2021 [29] and in Illinois, as documented by sequencing studies performed by the Illinois Department of Public Health (https://www.dph.illinois.gov/covid19/variants, accessed on 27 August 2021). Bioinformatic analysis of the deduced amino acid sequence of spike genes revealed eight distinct constellations of mutations in the spike as compared to the reference strain (accession ID: NC_045512.2) (Figure 2). Of the six *Alpha* lineage variants, four of them have the spike mutations identical to that of the original *Alpha* strain, while two samples each have an additional spike mutation. Similarly, of the four *Gamma* lineage samples, three harbored the spike mutations of the original *Gamma* strain, while one acquired an additional mutation in the cytoplasmic tail domain. We also detected individuals with other lineages: S4 and S6 with the 20G lineage, and S3 with the *Epsilon* lineage (*Epsilon*, 20C) that contains the concerning L452R mutation in spike [30]. All spike sequences contained the D614G mutation. The amino acid substitutions in the receptor binding domain (RBD) and N-terminal domain (NTD) have been associated with escape from vaccine-induced neutralizing activity [30,31]. We report that no specific variant was associated with more severe disease in healthy individuals or in solid organ transplant patients within this cohort. The variants we detected were representative of the viruses circulating in the Chicagoland area in April, 2021 (https://www.dph.illinois.gov/covid19/variants, accessed on 27 August 2021).

## 4. Discussion

We report that half (7/14) of the SARS-CoV-2 breakthrough infections in fully vaccinated people detected at our center over a four-week period occurred in immunosuppressed subjects, with the majority of these immunosuppressed subjects experiencing severe disease requiring hospitalization. Six of the hospitalized patients were SOT recipients, highlighting the vulnerability of these individuals. Combined with recent reports indicating that only a small percentage of transplant patients mount a detectable SARS-CoV-2 antibody response post vaccination [22,23,32,33,34], this finding suggests that some immunosuppressed individuals, particularly solid organ transplant recipients, may not generate robust protective responses after vaccination, making them more vulnerable to severe disease if they are infected by a SARS-CoV-2 variant. It remains to be determined whether the higher post-vaccine infection prevalence and associated disease severity observed in immunosuppressed individuals presented here is due to virus exposure in the setting of low or insufficient levels of neutralizing antibody elicited by the vaccine, evasion of vaccine-induced immune response by viral variants, or inadequate cell-mediated immune responses to virus infection. In line with the recommendations in early 2021, none of the immunosuppressed patients in this report were tested for anti-spike antibody response after completion of vaccination. Our current findings raise the question of whether immunosuppressed individuals should be tested for SARS-CoV-2 anti-spike antibody post-vaccination. A recent study indicates that immunization with a third dose of mRNA vaccine boosts the immune response in solid organ transplant recipients [35,36]. More studies are needed to determine the correlates of immunity required to protect from severe COVID-19, and the optimal approaches for eliciting these responses in SOT patients or others undergoing immunosuppressive therapies. 

One of the important issues for controlling the spread of variants is to determine if breakthrough infection is associated with high viral loads that may result in secondary spread. Previous studies reported that low viral loads were detected following vaccination [37,38]. In contrast, we detected relatively high viral loads (median C*t* of 19.6) even in non-immunosuppressed vaccinated subjects exhibiting asymptomatic or mild infection. Among three individuals who were asymptomatic or had mild symptoms, two of them had C*t* values of 18.8 and 19.5, respectively, which correlates with high viral loads. Asymptomatic or minimally symptomatic breakthrough infections may occur and go undiagnosed in healthy individuals. In the present study, infections were identified in subjects 1, 5, and 6 solely due to surveillance programs and post-exposure testing, similar to recent reports [4,13]. Breakthrough infection in vaccinated asymptomatic subjects, such as those described here, may contribute to the spread of SARS-CoV-2 in the population. Our study is in agreement with several recent reports documenting high viral loads in breakthrough infections in both symptomatic and asymptomatic individuals [7,12,39]. More studies are needed to determine the contribution of breakthrough infections in the spread of SARS-CoV-2 variants of concern. 

Another finding from this study is that we identified four distinct lineages of SARS-CoV-2 variants (Figure 1). These findings highlight that diverse virus lineages were co-circulating in the Chicago area (https://www.dph.illinois.gov/covid19/variants, accessed on 27 August 2021). The only spike mutation in common for all the variants was the D614G mutation, which arose in early 2020 and rapidly spread, such that this mutation is detected in viruses from around the world [40]. We note that subjects 1, 2, 3, 7, 8, and 9 harbored spike sequences from the *Alpha*/B.1.1.7 variant of concern lineage. The SARS-CoV-2 *Alpha*/B.1.1.7 lineage has been shown to be neutralized by convalescent serum from vaccinated individuals [18]. Three out of the six subjects infected with this variant of concern experienced mild disease, with the more severe disease seen in an elderly patient and solid organ transplant patients. The second variant of concern was the *Gamma*/P.1 lineage in subjects 4, 10, 11, and 12. This lineage contains the E484K and N501Y mutations that likely contribute to evasion of vaccine-induced immunity and has been associated with re-infection [31,41]. This variant was recently identified by rapid detection in Canada, highlighting the importance of surveillance testing in identifying variants of concern [42]. We also identified a variant containing the L452R mutation (S3) that has been reported to contribute to evasion of vaccine-induced immunity [30]. While this study was in progress, the SARS-CoV-2 *Delta* variant emerged as the predominant variant spreading in the USA. Recent studies report breakthrough infections and severe disease associated with the *Delta* variant in transplant recipients [14,43,44]. More studies are needed to identify approaches to elicit protective immune responses to emerging variants. 

Our study has limitations. The number of breakthrough infections is small (*n* = 14) over a one-month period at one medical center. The finding that six of the post-vaccination infections were in SOT recipients highlights the vulnerability of this group in our patient population. Our results are in agreement with the recent study of Brosh-Nissimov et al. that reported the characteristics of breakthrough infections in 152 vaccinated individuals identified from January to April 2021. They reported that 40% of the patients with breakthrough infections were immunocompromised (11% SOT patients), and that co-morbidities including hypertension and diabetes predisposed to severe COVID-19 [7]. Additional studies are needed to determine if SOT patients are vulnerable to specific variants, and if a subset of variants are associated with more severe disease. Another limitation of our study is that we did not have evidence of an immune response to the vaccine in our vaccinated subjects. Although the vast majority of healthy individuals respond to the vaccines authorized for emergency use, recent studies suggest that SOT patients may require a third immunization to elicit a detectable antibody response to spike protein [35,36]. Future studies will focus on evaluating the antibody response to vaccination in SOT and other immunocompromised patient populations. 

Overall, our results highlight the ongoing need for genetic analysis of breakthrough infections, and for developing strategies for reducing the spread of variants and protecting vulnerable populations from severe disease. Recent studies indicate that boosting of vaccinated individuals with vaccines designed to elicit immune responses to SARS-CoV-2 variants enhances neutralizing titers [45]. This approach is likely to reduce breakthrough infections and may lower viral loads in those who do become infected so that secondary spread of infection is also reduced. Importantly, screening for vaccine response in vulnerable populations, such as solid organ transplant recipients, and correlating the response with post-vaccination infections would begin to fill this gap in knowledge.

## 5. Conclusions

We identified SARS-CoV-2 breakthrough infections with variants of concern in vaccinated individuals, with solid organ transplant recipients and other immunosuppressed or elderly individuals at higher risk for severe disease. These results highlight the importance of ongoing monitoring of the spread of SARS-CoV-2 variants and the need for assessing the immune response to vaccination in immunosuppressed individuals.

## Figures and Tables

**Table 1:** Demographics of patients, clinical and virological findings.

	Subject	Sex	Age Range	Additional Risk Factors	Immunosuppressive Medication	Vaccine Type	Symptoms	Hospitalization	Viral Load (C*t*)	NextClade Lineage
Asymptomatic/mild	1	F	60	HCW	No	Pfizer	Rhinorrhea	No	18.8	20I/S:501Y.V1/VOC-alpha
	2	M	58	None	No	Pfizer	Chill, subjective fever	No	19.1	20I/S:501Y.V1/VOC-alpha
	3	F	48	Smoke	No	Pfizer	Weakness, congestion loss of taste/smell, fatique	No	20.9	20I/S:501Y.V1/VOC-alpha
	4	F	51	NASH	Yes	M/P	Headache, cough, rhinorrhea ageusia, anosmia	No	17.1	20J/S:501Y.V3/VOC-gamma
	5	F	37	HCW	No	Moderna	Asymptomatic	No	19.5	20G
	6	F	50	HCW	No	Pfizer	Asymptomatic	No	34.2	ND
hospitalized	7	F	81	Heart disease, CVA	No	J&J	Shortness of breath, cough	Yes	18.8	20I/S:501Y.V1/VOC-alpha
	8	M	65	SOT-kidney and heart	Yes	Pfizer	Diarrhea, myalgia, chills, fever, pneumonia	Yes	20.1	20I/S:501Y.V1/VOC-alpha
	9	M	55	SOT-kidney	Yes	Pfizer	Cough, acute hypoxic respiratory failure, sepsis	Yes, ICU, died	22.3	20I/S:501Y.V1/VOC-alpha
	10	M	70	SOT-liver	Yes	Pfizer	Cough, weakness, fever, dyspnea	Yes	19.6	20J/S:501Y.V3/VOC-gamma
	11	M	68	SOT-lung	Yes	Moderna	Acute hypoxia, acute pneumonia, hemoptysis	Yes, ICU	21.4	20J/S:501Y.V3/VOC-gamma
	12	F	60	SOT-lung	Yes	Moderna	Shortness of breath, fever, chills, body aches, hypoxia	Yes, ICU	15.7	20J/S:501Y.V3/VOC-gamma
	13	M	65	SOT-liver	Yes	Pfizer	Diarrhea, nausea, weakness cough, dyspnea	Yes	22.1	20C/epsilon
	14	F	76	None	No	Pfizer	Fever, chills, acute respiratory failure	Yes, ICU	18.3	20G

Abbreviations: HCW: healthcare worker; NASH: non-alcoholic steatohepatitis; CVA: cerebrovascular accident; SOT: solid organ transplant; Pfizer: BNT162b2; Moderna: mRNA-1273; M/P: mRNA vaccine type not provided; ICU: intensive care unit; Ct: cycle threshold; ND: not determined; VOC: variant of concern.

## Data Availability

The complete genomic sequences of the thirteen SARS-CoV-2 variants were deposited in the GISAID database with the following accession numbers: EPI_ISL_3048070, EPI_ISL_3048060, EPI_ISL_3048071, EPI_ISL_3048061, EPI_ISL_3048062, EPI_ISL_3048063, EPI_ISL_3048064, EPI_ISL_3048065, EPI_ISL_3048066, EPI_ISL_3048067, EPI_ISL_3048068, EPI_ISL_3048069.
